# Osteoarthritis in the XXIst Century: Risk Factors and Behaviours that Influence Disease Onset and Progression

**DOI:** 10.3390/ijms16036093

**Published:** 2015-03-16

**Authors:** Giuseppe Musumeci, Flavia Concetta Aiello, Marta Anna Szychlinska, Michelino Di Rosa, Paola Castrogiovanni, Ali Mobasheri

**Affiliations:** 1 Department of Biomedical and Biotechnological Sciences, Human Anatomy and Histology Section, School of Medicine, University of Catania, Via S. Sofia 87, 95123 Catania, Italy; E-Mails: flaviayellow@tiscali.it (F.C.A.); mszychlinska@unict.it (M.A.S.); pacastro@unict.it (P.C.); 2 Department of Biomedical and Biotechnological Sciences, Pathology Section, School of Medicine, University of Catania, 95123 Catania, Italy; E-Mail: mdirosa@unict.it; 3 The D-BOARD European Consortium for Biomarker Discovery, Department of Veterinary Preclinical Sciences, School of Veterinary Medicine, Faculty of Health and Medical Sciences, University of Surrey, Guildford GU2 7XH, UK; E-Mail: a.mobasheri@surrey.ac.uk; 4 Arthritis Research UK Centre for Sport, Exercise and Osteoarthritis, Arthritis Research UK Pain Centre, Medical Research Council and Arthritis Research UK Centre for Musculoskeletal Ageing Research, University of Nottingham, Queen’s Medical Centre, Nottingham NG7 2UH, UK; 5 Center of Excellence in Genomic Medicine Research (CEGMR), King Fahd Medical Research Center (KFMRC), King AbdulAziz University, Jeddah 21589, Saudi Arabia

**Keywords:** osteoarthritis (OA), risk factors, diet, sedentary lifestyle, high intensity and ballistic sports

## Abstract

Osteoarthritis (OA) is a growing public health problem across the globe, affecting more than half of the over 65 population. In the past, OA was considered a wear and tear disease, leading to the loss of articular cartilage and joint disability. Nowadays, thanks to advancements in molecular biology, OA is believed to be a very complex multifactorial disease. OA is a degenerative disease characterized by “low-grade inflammation” in cartilage and synovium, resulting in the loss of joint structure and progressive deterioration of cartilage. Although the disease can be dependent on genetic and epigenetic factors, sex, ethnicity, and age (cellular senescence, apoptosis and lubricin), it is also associated with obesity and overweight, dietary factors, sedentary lifestyle and sport injuries. The aim of this review is to highlight how certain behaviors, habits and lifestyles may be involved in the onset and progression of OA and to summarize the principal risk factors involved in the development of this complicated joint disorder.

## 1. Introduction

**Figure 1:**
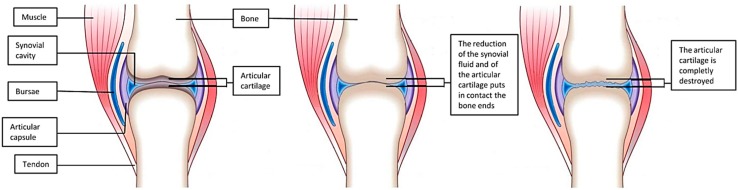
This figure shows the alterations that occur in the joints during the onset of osteoarthritis (OA).

Osteoarthritis (OA) is the most common type of arthritis. It is a crippling, late-onset and degenerative disease characterized by the loss of articular cartilage and synovial inflammation, leading to joint stiffness, swelling, pain and loss of mobility. For several decades, OA was considered as a wear and tear disease, leading to joint tissue destruction and disability. The widely held view was increased pressure or overload on weight-bearing joints, anatomical joint incongruence and fragility of articular cartilage tissue were the key predisposing factors. Nowadays, thanks to the advent of molecular biology and key discoveries in the field, OA is being redefined as a very complex and multifactorial disease (Figure 1). From the epidemiological point of view, because of the high percentage of people suffering from this disease and the increase in life expectancy, OA is now considered as one of the most significant causes of disability in the world. Currently, OA affects about half of the over 65 population with a greater percentage in women than in men after menopause (18% *vs.* 9.6%) [1]. Although OA mainly affects the joints of knees, hands and hips [2], it also results in alterations in other joint tissues such as ligaments, synovium and subchondral bone [3]. OA can broadly be classified in two different forms, primary and secondary. The primary or idiopathic OA, is a gene-dependent disease. Various studies have indicated a strong hereditary component in primary OA, probably due to its polygenic nature [4]. Secondary OA, also called post-traumatic OA, frequently occurs sometime after a traumatic event. Secondary OA is exacerbated by inflammatory and repair processes that occur after the initial traumatic insult and after surgery. Studies have reported the high risk of secondary OA in sportsmen and sportswomen engaging in high impact and ballistic sports such as football (soccer), rugby, basketball and downhill skiing. The latter for example depends on their great propensity for abnormal movements, especially knee-pivoting [5]. Today, a growing influence of technology in our daily life should be also evaluated as a possible risk factor for the onset of secondary OA. The increased use of tablets, smart-phones and mobile phones, but also keyboards and mice, can be considered a risk factor for hand and wrist OA.

Although primary and secondary OA are caused by different factors, the resulting pathology is the same: a degenerative phenomenon, complicated by inflammatory reactions [2,6] (Figure 1). Even though different studies actually refer to several ordinary risk factors for OA, like genetic and epigenetic predisposition, age, sex, injury and mechanical stress [6], there is a common belief that also diet and lifestyle can influence the appearance of arthritis. Susceptibility and predisposition to OA depends then on the association of various risk factors.

Specifically, the onset of knee OA has been mainly associated with excess weight, obesity, female gender and previous knee injury. Diabetes is also considered a risk factor for the progression of knee OA. On the contrary to what is thought, in patients with knee pain, only 5.1% of cases were due to previous knee injury, instead 24.6% was related to excess weight or obesity. Clinicians should take into account these data to identify and manage patients at risk of developing or increasing knee pain. The management of obesity, in particular, needs to be a major target for prevention of development of knee pain. There is, however, limited evidence regarding factors such as the influence of co-morbidities and socio-economic status and, therefore, further research needs to focus on these risk factors rather than those for which extensive evidence already exists [7].

Nowadays, the most innovative approach in investigation and treatment of joint disorders and OA is represented by tissue engineering strategies. Indeed, this technique, applied to chondrocytes and mesenchymal stem cells (MSCs), isolated from different sources such as adipose tissue [8,9], could be successfully applied in repairing articular cartilage lesions [10] and, ultimately, the research in this field focuses more and more on this type of therapeutic approach. Tissue engineering consists of a use of a combination of cells, engineering, and materials associated with biochemical and physio-chemical factors in order to improve or replace biological functions. Cartilage could benefit from several improvements through the use of tissue engineering. This depends on some characteristic properties of the tissue itself; in fact, it is avascular, aneural and alymphatic, and it is constituted by just one cell type, the chondrocytes [11]. For this reason once injured, it is not able to repair itself and it has to be replaced.

## 2. Predisposition

**Figure 2:**
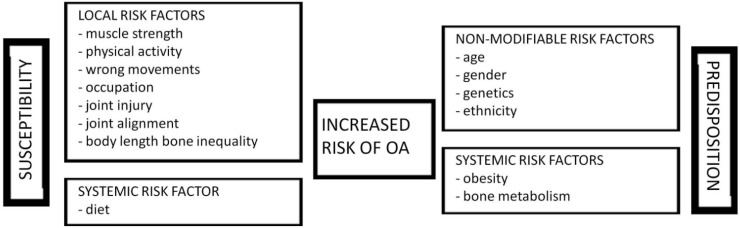
The scheme represents the major risk factors that lead to the increased susceptibility and predisposition to develop OA.

The most important predisposing factors for OA are shown below. These include genetic and epigenetic factors, age, gender and ethnicity. These all represent non-modifiable risk factors (Figure 2).

### 2.1. Genetic and Epigenetic Predisposition

A number of recent studies discovered the presence of over 80 gene mutations involved in the pathogenesis of OA [4], among which the most relevant one is a single nucleotide polymorphism. This one, called rs143383 and located in the 3' untranslated region (3'UTR) of the growth and differentiation factor 5 gene (GDF5) [2], is responsible for the development, maintenance and repair of synovial joints [12]. Genes for Vitamin D receptors (VDR) and insulin-like growth factor 1(IGF-1) also seem to be involved in the patho-physiologic pathways of OA [13]. The first one, whose locus is localized near that for type II collagen, controls bone density [13]. Recently, also genome-wide association scanning (GWAS) identified a correlation between hand OA and the decreased expression of ALDH1A2, an aldehyde dehydrogenase 1 family, member A2 gene [14]. Besides the great number of genes partially involved in OA, and the polygenic nature of the disease, it seems also interesting that OA does not actually show any high-impact locus, but a mix of different genes expressing proteins that play a role in the manifestation of OA.

The term epigenetic means all the heritable changes that occur in phenotypes without any alteration in genotypes, therefore in the primary DNA sequence. Unlike genetic modifications, the epigenetic ones are more rapid and the cells use them as a response to changes in their microenvironment. The epigenetic patterns, mediated by DNA methylation, histone modifications and non-coding RNAs, respond to external stimuli, cell proliferation and differentiation [2]. As a result of these modifications, the expression of some genes can be modulated by introducing genetic susceptibility [2]. The most evident alterations that appear to be under epigenetic regulation are those in MMP-13 and IL-1 β promoter. MMP-13 is an enzyme involved in the cartilage matrix destruction in OA. Recent studies demonstrate that this alteration depends on a demethylation of CpGsites in the MMP-13 promoter [15]. In human chondrocytes, three different alterations are associated with increased levels of MMP-13 gene expression: a site at −104, −110 and −299 bp [16]. Another study shows similar sites in the IL-1 β promoter.

### 2.2. Age, Cellular Senescence, Apoptosis and Lubricin

As previously described, OA is a degenerative disease. Although several studies confirm the high correlation between age and OA, the real mechanism has still not been definitively identified. The correlation probably depends on some modifications that occur in the normal structure of the joints and that affect chondrocytes and the extracellular matrix, which result in a decreased capacity of the joint to adapt to different insults, both chemical and mechanical [17]. In fact, the chondrocytes have a limited number of replications allowed during their lives (approximately 30–40 divisions). This replication number is also known as the “Hayflick limit” [18]. This phenomenon takes place towards the end of the cell-cycle progression. Many hypotheses suggest that in each cell division a fragment of the normal structure of the chromosome gets lost. This part, that originates from a structure of the same chromosome, called telomere, becomes shorter after every cell cycle. This mechanism protects the DNA coded sequences and consequently prevents the loss of the genetic code. Furthermore, the length of the telomere could be used as a marker for replicative senescence [1], allowing researchers to evaluate the number of cell cycles still remaining. It is a common belief that the senescence of the chondrocyte is the main factor responsible for the development and progression of OA, because the senescent cells lose the capacity to maintain and repair the extracellular matrix (ECM) of cartilage [19]. In fact, ECM undergoes changes with age, such as alteration in composition and structure of proteins and proteoglycans, surface fibrillation, increase of cross-linking in collagen and the resulting reduction in tensile strength [20]. This results in an increased risk to compromise the tissue after a mechanical or load-induced stress [19]. Moreover, the aging process involves a significant decrease in the number of chondrocytes in articular cartilage, which undergo apoptotic death and this correlates directly with the degree of cartilage damage [21]. Although many authors have reported the apoptotic chondrocytes in OA cartilage, only a few have examined the correlation between apoptosis and aging in normal cartilage and just one study in rat cartilage found evidence of increased apoptosis with aging [22]. Under normal physiological conditions, chondrocytes maintain equilibrium between synthesis and degradation of ECM components, regulating in this way structural and functional integrity of cartilage [23]. Apoptosis features, detected in OA chondrocytes, are associated with matrix degradation and calcification, that suggest an important role of cell death/survival mechanisms in OA pathogenesis. Moreover, apoptosis has also been strongly correlated with the severity of cartilage damage and matrix depletion in humans [24]. A hypothesis is that apoptosis occurring in chondrocytes could be secondary to cartilage degradation as cell matrix interaction is vital for chondrocyte survivability. Chondrocyte survivability is mediated, indeed, by integrins, responsible for the connection of extracellular matrix components such as collagen, laminin and fibronectin, to various intracellular cytoskeletal proteins [25]. Loss of this adhesion may induce chondrocytes to apoptosis. It is likely that degeneration of chondrocyte matrix is due to direct injury to the cartilage, causing biochemical changes or loss of ECM components [26,27]. The extent of chondrocyte apoptosis is also correlated with fibronectin expression. The latter represents one of the key ECM molecules involved in communication between cartilage cells and their surrounding matrix. Its up-regulation is associated with the severity of articular cartilage damage [27]. Thus, decreased expression or availability of important matrix macromolecules in cartilage is sufficient to induce chondrocyte apoptosis and cause matrix degeneration [26,27]. A chondroprotective agent of articular cartilage is represented by the mucinous glycoprotein product of the proteoglycan 4 (PRG4) gene, called lubricin or superficial zone protein (SZP) [28]. Lubricin could be used to determine the onset and the progression of the disease [29]. This chondroprotective glycoprotein plays different roles in the cartilage tissue, such as lubrication of cartilage surfaces [30], prevention of cartilage wear and synovial cell adhesion. It also reduces the coefficient of friction on the articular cartilage surfaces [31,32,33,34]. Data from several studies revealed that recombinant lubricin, administered to OA animal models, seems to protect articular cartilage and prevent the progression of the disease [35], suggesting its possible use as an innovative therapeutic treatment for OA.

### 2.3. Gender

Several epidemiologic studies of OA suggest the relevant difference between pathological pathways occurring during the onset of this disease in males and females. Women usually show a higher prevalence of OA in the hand, foot and knee than men [36]. Making a comparison in 50-year-old aged patients, OA is more present in men than in women. Afterwards, generally after menopause, the prevalence of OA in women significantly increases [37,38]. In fact, approximately 9.6% of men and 18% of women show symptomatic OA [1]. This observation suggests that hormonal factors could influence progression and development of the disease. The disparities may also be dependent on the difference in the structure of bones and ligaments, such as strength and alignment, laxity of ligaments or just a reduced volume of cartilage in women compared with that of men.

### 2.4. Ethnicity

The studies about OA are in conflict with the correlation between ethnicity and OA. Some studies conducted by the National Health and Nutrition Examination Survey I (NHANES I), suggested that African-American women are more likely to develop knee OA than men and white persons [13], while the Johnston County Osteoarthritis Project (JCOAP) evidenced no ethnic differences [39,40]. The results are quite the opposite in hip OA, where NHANES did not reveal differences [41] whereas the JCOAP did [42]. It probably depends on lifestyle or socioeconomic factors, body mass index, and even genetic factors [43].

## 3. Susceptibility: XXIst Century Lifestyles, Behaviors and OA

Increased cost of living and life expectancy, force more and more people to reduce the time that they used to dedicate to themselves in order to take care of their health. As a consequence, people dedicate less time to sports activity and/or to good and healthy meals. Some of the most important susceptibility factors for OA development are indicated below. These risk factors are represented by the habits and behaviors affecting our daily lives, including food (diet and obesity), physical activity (sedentary lifestyle or extreme competitive sports) and technology (Figure 2).

### 3.1. Diet and Obesity

**Figure 3:**
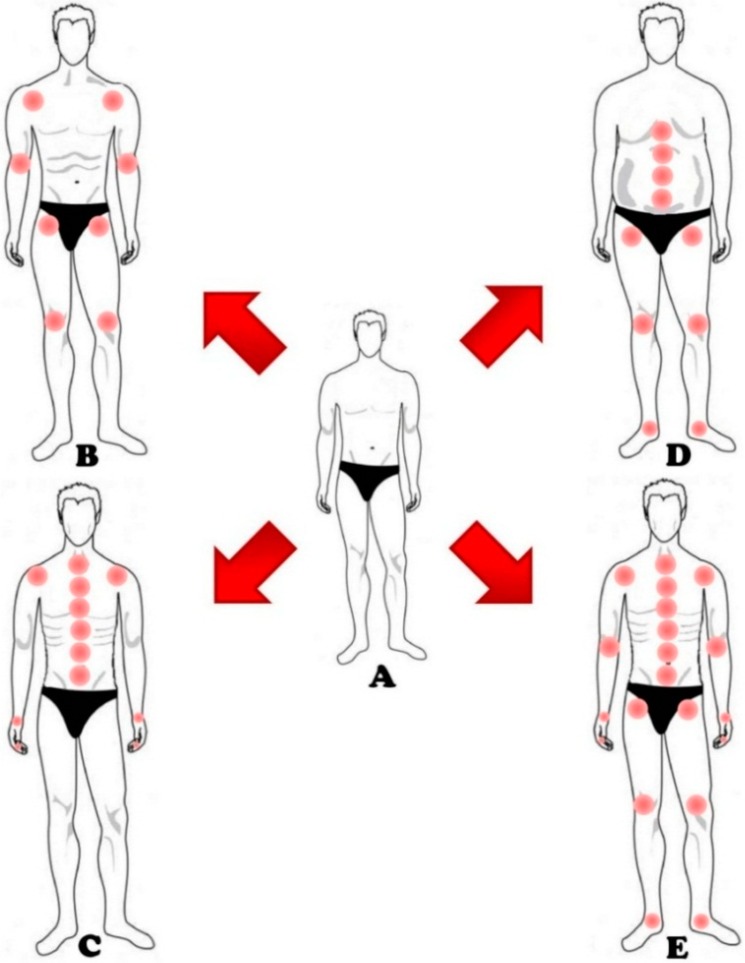
This figure shows 5 different lifestyles. (**A**) Pictograph A shows a person who leads a healthy life choosing a controlled diet, rich in nutrients and vitamins, and moderate physical activity. This subject reduces the risk to develop OA, both in cartilage alterations and inflammation; (**B**) Pictograph B shows a sportsman. In this case, the extremely forced physical activity, wrong movement, and direct joint impact or torsional load, lead to an increased joint risk. This risk is mostly located in the joint of the shoulders, arms, hips and knees; (**C**) Pictograph C shows a person who leads a sedentary lifestyle. Here, little physical training is responsible for muscle weakening and, consequently, to wrong posture. The major risk is visible in the joints of the spinal column and shoulders. Furthermore, the use of smart phones and computers increases the risk of OA in the joints of hands and wrists; (**D**) Pictograph D shows an obese subject. Here, the increased body weight lies heavily on the joints of the spinal column and of the lower limbs, such as hips, knees and ankles; (**E**) Pictograph E shows a subject not consuming a proper diet. The loss of vital nutrients increases the risk of developing OA in most of the joints of the subject itself.

Many studies suggest that diet and nutrition influence our lives; everything we eat determines the initiation of different processes in the body. The diffusion of fast food and the excessive consumption of snacks are strictly becoming predominant in daily life. This diet is mainly constituted by preservative and harmful substances, such as animal fat and glucose excessive concentration and lack of nutritional factors, such as vitamins and nutrient minerals essential for our body. Moreover, the bad dietary habits may predispose people to obesity, which is responsible for both metabolic destroying processes affecting the cartilage and overload of the joints, especially hips and knees [44] (Figure 3).

A recent study was conducted to determine the association of OA with dietary factors, such as quantity and quality of nutrient intake. Low intake of vitamin D and vitamin C is a possible risk factor for knee OA, while certain food groups, such as milk and dairy products, meat and poultry are beneficial for knee OA. Thus we could affirm that nutritional imbalance, combined with endocrine abnormalities, may be involved in the pathogenesis of OA [45]. OA is increasingly considered as a systemic disease, especially in terms of a possible relationship to metabolic disorders linked to obesity. Obesity is one of the risk factors for hip or knee OA, since mechanical overload on weight-bearing joints activates chondrocytes and accelerates cartilage degeneration [46]. However, the association between obesity and OA in non-weight-bearing joints suggests a more complex aetiology for obesity-induced OA. Anyway, to date, this process is not fully understood. It has been proposed that metabolic factors and their clustering in metabolic syndromes, such as inflamed adipose tissue and dyslipidaemia, could play a crucial role in obesity-induced OA [47,48,49,50,51]. Indeed, several studies affirm that there is an increased risk of OA of the knee and other joints associated with metabolic risk factors such as oxidative stress, endothelial dysfunction and leptin dysregulation that are considered part of the metabolic syndrome [13,52,53]. Apart from therapeutic approaches aimed at reducing pain, actually there is no treatment that targets and inhibits the progressive degenerative structural changes. Recent data suggesting that OA shares a similar biochemical and inflammatory profile to the metabolic syndrome may be helpful in detecting OA early before irreversible damage to the joint has occurred [54,55,56,57].

#### 3.1.1. ROS and Vitamins

The onset of OA, as with many other common age-related diseases, seems to be associated with the continuous exposure to oxidants [58]. The reactive oxygen species (ROS), which are mostly produced by chondrocytes, may damage cartilage collagen and synovial fluid, by reducing its viscosity [58,59,60,61,62]. It seems relevant that the intake of some vitamins, which have antioxidant properties, can reduce the level of ROS and, at the same time, the outcome and the development of the disease. This can be related to Vitamin C and K. Low levels of Vitamin C increase the risk of knee OA [63], while high Vitamin C intake seems to reduce both the progression of radiographic knee OA and pain [64]. On the other hand, low plasma levels of Vitamin K (also known as phylloquinone), which normally regulates mineralization of bone and cartilage, increase the prevalence of osteophytes and joint space narrowing in the hand and osteophytes in the knee [65]. Theories about the role played by Vitamin D in the OA progression are controversial, in spite of the fact that most of its physiological functions take place in the bones. Previous studies discovered an increased incidence firstly of hip OA and then of knee OA at low or moderate levels of vitamin D [63,66,67]. Conversely, recent trials demonstrated no significant correlation between cartilage loss and low levels of vitamin D [64,68]. Moreover, a recent study reported a beneficial effect of the assumption of extra virgin olive oil, rich in antioxidants such as Vitamin E, A and K, in rats suffering from OA, underlining its possible application in the preventive treatment of this disease [28].

#### 3.1.2. Glucose Concentration

There is evidence suggesting that metabolic factors such as type 2 diabetes and elevated glucose concentration are particularly connected with OA development and progression [51]. In particular, the advanced glycation end-products (AGEs) in cartilage collagen seem to be related both with senescent cartilage matrix and with reduced function of chondrocytes. This alteration may depend on the expression of the AGE receptors (RAGE), which seems to be increased both in aging and in OA [69,70]. Both matrix and chondrocytes tend to accumulate AGEs, causing, in matrix, an increased cross-linking, stiffness, reduced strength of the joints and finally making the cartilage more susceptible to failure [71]. In chondrocytes, the function seems to be altered due to the decreased anabolic activity. As a result, the presence of AGEs associated with the expression of RAGEs in the cartilage collagen results in increased production of MMPs and in modulation of the chondrocyte phenotype to hypertrophy and OA [72,73].

#### 3.1.3. Adipokines

**Figure 4:**
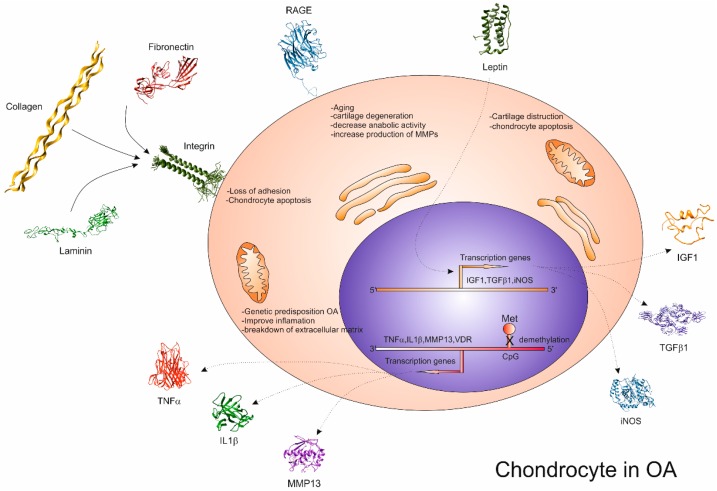
The OA-associated molecules, including IL-1β, TNF-α, RAGE, leptin, IGF-1, TGFβ1, iNOS, MMP13, laminin, fibronectin, integrin and collagen are involved in chondrocyte activation. These molecules contribute to the pathogenesis of OA by destroying the cartilage in the joints or serving as the substrates for extracellular matrix destruction (*i.e*., laminin, fibronectin and collagen type II).

**Figure 5:**
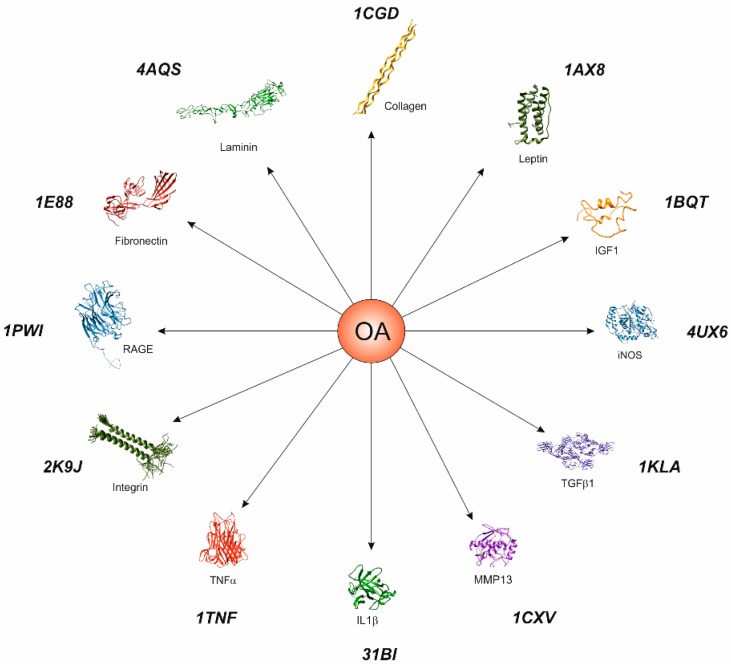
Target genes involved in OA. Molecular graphics and analyses were performed with the UCSF Chimera package. Chimera is developed by the Resource for Biocomputing, Visualization, and Informatics at the University of California, San Francisco.

As mentioned above, obesity represents a common disease usually associated with OA. Several studies suggest that overweight people are more inclined towards the development of OA than normal-weight people [50,52,74,75]. In 1994, Carman W.J. and colleagues suggested the possible link between OA and obesity [76], due to some metabolic and inflammatory systemic effects explaining the presence of symptomatic hand OA. Recent advances in the physiology of adipose tissue suggest that the relationship between obesity and OA may be caused by some systemic factors. In fact, the presence of OA can be verified not only in joints that are more affected by excess weight, such as knees and hips, but also in non-weight bearing joints such as hands [77]. In fact, the onset of OA and its symptoms may be prevented more by the loss of body fat than by weight loss. The systemic factors that could influence the onset of OA, acting as a metabolic link between obesity and OA, may be represented by the adipokines, such as leptin, adiponectin, resistin, and visfatin. These adipokines mediate some important functions in the metabolic pathways, such as lipid and glucose metabolism, insulin sensitivity, and other physiological functions, such as reproductive functions, blood pressure regulation, bone formation, and angiogenesis [78]. The connection between OA and adipokines derived from several recent hypotheses that classified OA as a systemic disorder caused by an alteration in lipid homeostasis [79]. The most important of the adipokines that seems to be involved in the onset of OA, is represented by leptin. This one may influence both growth factor synthesis and chondrocyte anabolism and catabolism through the activation of Signal Transducers and Activators of Transcription (STATs) type 1 and 5, but not STAT 3 [80]. Articular tissues, with strong structural and biochemical changes, show a less regulated leptin expression in comparison with normal tissues [81] and, in particular, the level of leptin expression is proportional with the grade of cartilage destruction, and, consequently, those of growth factors (insulin-like growth factor I and transforming growth factor β-1). Despite the beneficial role that leptin may have on cartilage synthesis, an excess of leptin may reduce the extracellular matrix synthesis leading to increased susceptibility of the joints to lesions [82]. The cartilage destroying mechanism of leptin could be explained by the association of this adipokine with proinflammatory cytokines, such as interleukin 1 (IL-1), resulting in increased nitric oxide (NO) production. It is known that NO interferes with chondrocyte function causing loss of cartilage matrix by apoptosis induction, MMP activation, and type II collagen synthesis inhibition (Figure 4 and Figure 5) [83].

### 3.2. Movements and Physical Activity

Moderate physical activity and normal mechanical joint loading is extremely important in a healthy joint. Moderate exercise promotes a correct movement of our body, preventing weakening of the joint and alterations in the articular cartilage [84]. Some data also suggest that, in damaged or old joints, physical activity can improve tribology and lubricative properties of articular cartilage. In rat models, this activity has been shown to promote lubricin synthesis and increase in synovial fluid, which results in decreased cartilage degradation processes if compared with sedentary adult rats, due also to the decreased expression of IL-1, cytokine, strongly associated with the OA physiopathology [85]. On the other hand, excessive physical activity, joint use and mechanical stress could be associated with an increased risk of OA [86]. Our body, in spite of some mechanical defense structures (the thickness of articular cartilage, the bones located near the joint and the neuromuscular control of the joint) and sport organization training programs, is still subjected to joint injuries. One of the most important load-supports is granted by the high water content of articular collagen–proteoglycan matrix [87]. This interstitial fluid helps in supporting the joint load, reducing proteoglycan matrix stresses and friction at the articular surfaces [88,89,90]. Changes in cartilage composition and elevated water content can affect the joint load, causing cartilage deformation [91,92]. In fact, during cartilage loading, water (aquaporins expression) is gradually squeezed out of this highly hydrated tissue, causing strains in tissue, cells, and nuclear structures [93,94]. 

In OA, two aspects must be considered: repetition of movements and their wrong execution. The first aspect reflects the development of OA in people that, for their work or occupation, are forced to do repetitive movements. In this case, the risk of developing localized OA is doubled in comparison with people whose employment does not require physical activity and repetition of the same movements [95]. The second aspect concerns the wrong movements that could be done during sport activities especially in competitive sports or in those with an increased acute and direct joint impact or torsional load (such as baseball, soccer, skiing, or rugby) [96]. In these circumstances, the knee remains the most commonly damaged joint, especially for the rupture of the anterior cruciate ligament (ACL) and menisci [97]. The ACL is usually associated with the damage of articular cartilage, subchondral bone, collateral ligaments and menisci [98], which often leads to the development of secondary or post-traumatic OA (Figure 3) [99,100].

It must also be considered that the most common bad habits can influence the onset of OA. It is already inevitable that technology and its use is strongly increasing in daily life. Everyone now has at least a smartphone, a computer and/or a tablet. Some previous studies demonstrate that occupations or activities that require manual dexterity are associated with hand OA [101]. It is certain that use of smart-phones and computers involves an excessive stress on the joints of the hand. Generally the three most affected joints are those at the bottom of the thumb, those on the end of the fingers and the small joints within the fingers. It is usually associated with pain, site-specific disability, and reduced fine precision pinch [102]. It must be also considered that technology is becoming predominant not only in common adult life as an aid during work or occupation but also with children who use it. The age of “digital natives” decreases with time so that ever-younger children now use these technologies, reducing the time that they used to dedicate to other activities. As children grow, their initial approach to the technology, from the discovery of it, evolves into its daily use. Video games, PlayStations and Game Boys, but also mobile-phones, especially through the increasingly common habit of texting instead of calling, or keeping up with friends on social networks, tends to bond children and adolescents to the technology, increasing the risk of developing hand OA. In these circumstances, the repetitive movements they make with mobile phones and joysticks lead them to develop hand articular dislocations more easily, as their bones and joints are under development and therefore more vulnerable. In this context, the reduced physical activity leads also to less strength of their muscles and to an increased predisposition to have the wrong posture. This factor results in weakening of the joints, as well as the increased risk of becoming obese, that finally may act as a predisposing factor to the onset of OA. The studies focusing on this subject are very rare, and the real link between hand OA and technology cannot be proven, but, undoubtedly, several studies have to be made to investigate more in this field. 

## 4. Conclusions

Daily life and habits, work, diet and physical activity drastically influence our body. The body is in an extremely controlled balance, where every little change may be responsible for several alterations. Our diet and moderate physical activity are necessary to control this equilibrium and to avoid modifications also in our joints. Diets intended to achieve weight loss, physical activity, and physiotherapy are extremely important and should be encouraged as they reduce pain and improve joint function in OA but, at the same time, they are also relevant for other common and chronic diseases like diabetes and hypertension. A controlled diet, full of all the required nutritional factors, especially vitamins, may reduce the oxidative stress responsible for inflammation, and the decrease of fat and glucose concentration may eliminate some of the predisposing factors to obesity and then to OA. Physical activity and physiotherapy, when moderate and without forcing the body itself, are important to the muscles and to the joints. Also the hydration equilibrium is important, and if it is maintained, correct functioning of the joints occur. Furthermore, reducing the time spent using cell phones and other electronic devices and increasing those in which physical activity and taking care of ourselves is required, could surely result in a better health of joints and of entire body. In conclusion, we could state that taking care of our body is fundamental in order to prevent several diseases such as OA.
